# Real-world retrospective cohort study evaluating the tolerability of immune globulin intravenous 10% (BIVIGAM^®^)

**DOI:** 10.3389/fimmu.2025.1687588

**Published:** 2025-12-16

**Authors:** Lucinda J. Van Anglen, Quyen Luu, Kevin P. Rosenbach, Richard F. Herrscher

**Affiliations:** 1 Healix Infusion Therapy, LLC., Sugar Land, TX, United States; 2 Central Georgia Infectious Disease, Macon, GA, United States; 3 Naples Allergy Center, Naples, FL, United States; 4 AIR Care, Dallas, TX, United States

**Keywords:** intravenous immunoglobulin (IVIG), primary immunodeficiency (PID), BIVIGAM^®^, tolerability, real-world evidence (RWE)

## Abstract

**Introduction:**

Intravenous immune globulin 10% liquid (IVIG 10%) is used in the treatment of primary immunodeficiency (PID). Originally approved in 2012 and then voluntarily withdrawn in 2016, IVIG 10% (BIVIGAM^®^) underwent manufacturing process improvements by a new manufacturer and was reintroduced to the US market in 2019. The purpose of this real-world study is to assess the tolerability of BIVIGAM in an outpatient setting.

**Methods:**

An observational, retrospective analysis was performed using a random sample of patients who initiated BIVIGAM from 8/2021-5/2022 in 15 US outpatient physician office infusion centers. Patient data collected from electronic medical records included baseline characteristics, BIVIGAM treatment details, adverse events (AEs) and clinical laboratory data for 6 months following initiation.

**Results:**

A total of 60 patients treated with BIVIGAM were included. The mean age was 74 ± 8.2 years, 82% were female, and 90% had prior experience with immune globulin therapy. Treatment diagnoses were PID-related in the majority of patients (58,97%).There were 346 BIVIGAM infusions (mean of 5.8 ± 2.6 infusions per patient, mean dose of 492 ± 200.8 mg/kg),titrated upward over a mean of 80 ± 27.9 minutes to a final maximum mean infusion rate of 158 ± 28.8 mL/hr. Pre-medications were used in 83% of patients during 266 infusions. Forty-six AEs were patients (38.3%), resulting in an overall AE rate per infusion of 0.13 per patient year.

**Discussion:**

BIVIGAM was well-tolerated within this real-world outpatient setting. Infusion-related adverse reactions were low. This study provides the largest real-world evaluation of reformulated BIVIGAM in outpatient practice.

## Introduction

Intravenous immunoglobulin (IVIG) is used for treatment of primary immunodeficiency (PID) that comprises a group of mostly inherited disorders that weaken the immune system. Individuals with antibody deficiencies are susceptible to recurrent bacterial and viral infections, among other health problems. IVIG has been shown to be safe and effective in reducing serious infections in the treatment of a variety of PIDs. Other observed benefits include reduced antibiotic use, reduced incidence of mild infections, fewer hospitalizations, fewer school/work absences, and improved quality of life (1–4).

In April 2006, the American Academy of Asthma, Allergy and Immunology published evidence-based guidelines on indications for IVIGs for treatment of primary immunodeficiencies (1). IVIG has subsequently been approved by the FDA for chronic inflammatory demyelinating polyneuropathy (CIDP), chronic lymphocytic leukemia (CLL), immune thrombocytopenic purpura (ITP), multi-focal motor neuropathy (MMN), dermatomyositis (DM), Kawasaki Disease, Pediatric HIV infection, and prevention of graft versus host disease (GVHD) (5–8).

BIVIGAM^®^ is a human immune globulin intravenous 10% liquid (IVIG 10%) indicated for the treatment of patients with PID 2 years of age and older. This includes, but is not limited to, the humoral immune defect in common variable immunodeficiency (CVID), X-linked agammaglobulinemia (XLA), congenital agammaglobulinemia, Wiskott-Aldrich syndrome (WAS), and severe combined immunodeficiencies (SCID) (9).

A multicenter, open-label, non-randomized Phase III trial of BIVIGAM (NCT00538915), conducted between 2007 and 2009, evaluated 63 patients with PID receiving IVIG replacement therapy and demonstrated the product’s efficacy, safety, and tolerability (10). Two of 58 patients in the intent to treat population experienced serious bacterial infections, meeting the study endpoint of 0.037 acute bacterial infections per person-year. Fifty-nine subjects (94%) had an adverse reaction at some time during the study. The most common adverse events (AEs) observed in the clinical trial were headache (32 subjects, 51%), sinusitis (24 subjects, 38%), fatigue (18 subjects, 29%), upper respiratory tract infection (16 subjects, 25%), cough (14 subjects, 22%), diarrhea (13 subjects, 21%), bronchitis (12 subjects, 19%), pyrexia (12 subjects, 19%), and nausea (9 subjects, 14%) (9, 11).

BIVIGAM was originally developed by Biotest Pharmaceutical Corporation (Biotest, Dreieich, Germany) and, over time, was found to have multiple manufacturing inconsistencies and quality control problems that led to FDA observations resulting in FDA issuing a warning letter to Biotest Pharmaceuticals Corporation in November 2014. BIVIGAM was voluntarily withdrawn from the US market in December 2016 (12). ADMA Biologics, Inc. acquired the Biotest Therapy Business Unit in June 2017 and initiated a data driven approach for optimizing the IVIG manufacturing process that would bring BIVIGAM into complete FDA compliance (13). BIVIGAM was approved in May 2019 for re-introduction into the US commercial marketplace (14, 15).

Given the manufacturing changes and re-introduction of the product into the US market, assessment of post-marketing clinical experience is lacking. We hypothesized that reformulated BIVIGAM would demonstrate favorable tolerability with a low rate of infusion-related adverse events in adult patients with PID treated in outpatient settings. The purpose of this study was to evaluate the real-world tolerability of BIVIGAM.

## Methods

### Study design

This was a real-world, retrospective, observational, single-arm cohort study that included BIVIGAM patients seen over a six-month period in 15 US physician office infusion centers that utilize immunoglobulin therapy. Treatment indications, dosing, and frequencies were per physician discretion. Details regarding dosing, administration, IgG concentrations, pertinent labs, and adverse events while on BIVIGAM therapy were obtained at each visit.

Study data were extracted retrospectively through electronic health records, including standardized physician orders, infusion visit records, laboratory data, nursing assessments, provider assessments, progress notes, and on-call reports. Data were collected for each study patient for up to six months or until treatment discontinuation, whichever came first. The study protocol was approved by an independent institutional review board (Brany IRB, #22-12-676-1297, Melville, NY). The committee waived informed consent because the research involved only retrospective analysis of de-identified data in accordance with national regulations.

### Study population

The study population consisted of adult patients (≥18 years) who had received at least one dose of BIVIGAM. Patients were prescribed BIVIGAM by providers across various specialties (e.g., immunology, allergy, asthma, infectious disease, neurology, or rheumatology). Therapy was provided as ordered in the office through monitored infusions.

### Data collection

Data collection included patient demographics of age at BIVIGAM initiation, sex, weight, height, and body mass index (BMI). Clinical characteristics included comorbid conditions, primary diagnosis, and previous Ig therapy experience. Therapy details collected were the BIVIGAM regimen, dose (mg/kg), total dose, frequency, total volume of infusion, infusion maximum rate, infusion ramp time, total infusion time, premedication and hydration dosing details. Vital signs were collected at pre-infusion, mid-infusion, and post-infusion. Laboratory values were assessed for white blood cell count, hemoglobin, hematocrit, serum creatinine, blood urea nitrogen, liver function tests (aspartate aminotransferase, AST; alanine transaminase, ALT; and alkaline phosphatase, AP). AEs were defined as those temporarily associated side effects occurring during or within 72 hours of completion of BIVIGAM infusion, such as hypotension (defined as a 30 mmHg drop in systolic blood pressure from baseline), headache, fever, chills, myalgia, arthralgia, nausea, vomiting, back pain, abdominal pain, infusion site reactions, and others confirmed by the provider. Tolerability was measured as the incidence of AEs occurring during the study period to include headache, fever, chills, myalgia, arthralgia, nausea, vomiting, back pain, abdominal pain, infusion site reactions, or other reported AEs.

### Statistical analysis

Due to the availability of data and real-world nature of this study, all available patients who met inclusion criteria were included in the study. No sample size calculations were completed. Frequencies and percentages were utilized to assess maximum infusion rate and to describe AEs. The overall rate of AEs was calculated per infusion. Study variables were assessed using descriptive statistics. For continuous study variables, means, standard deviations (SDs), medians, interquartile ranges (IQRs), and minimum and maximum values were utilized. For categorical data, frequencies and percentages were tabulated. Missing data were not imputed; all available data were analyzed and presented as observed. Differences between baseline and follow-up laboratory parameters and blood pressure values were assessed using paired t-tests in GraphPad Prism version 10. A p-value <0.05 was considered statistically significant.

## Results

A total of 60 patients initiated BIVIGAM between August 2021 and May 2022 at 15 physician office infusion centers and were followed for six months. Centers were geographically diverse with 12 sites being in the South and 3 sites in the Midwest. **Table 1** provides the baseline demographic and clinical characteristics of the study cohort. The mean ± SD age of study patients was 74 ± 8.2 years with the majority of study patients of female sex, 49 (82%). The most common comorbidities were hypertension (n=33, 55%), cardiac comorbidities (n=29, 48%), gastroesophageal reflux disease (n=27, 45%), and asthma (n=24, 40%). The majority of study patients, 58 (97%), had a treatment diagnosis of PID, including 29 (48%) CVID, 18 (30%) nonfamilial hypogammaglobulinemia, and 11 (18%) selective deficiency of immunoglobulin G subclasses). Two patients (4%) had primary diagnoses not related to PID (one patient with dermatomyositis and the other with CLL).

**Table 1:** Baseline demographics and clinical characteristics.

Parameter	All patients N=60
Age in years, mean ± SD	74 ± 8.2
Female sex, n (%)	49 (82)
Body mass index in kg/m^2^, mean ± SD	27 ± 5.7
Common comorbidities, n (%)	
Hypertension	33 (55)
Cardiac comorbidities*	29 (48)
Gastroesophageal reflux disease	27 (45)
Asthma	24 (40)
Primary diagnosis, n (%)	
PI diagnosis	
Common variable immunodeficiency	29 (48)
Nonfamilial hypogammaglobulinemia	18 (30)
Selective deficiency of IgG subclasses	11 (18)
Chronic lymphocytic leukemia	1 (2)
Dermatomyositis	1 (2)
Ig treatment experience, n (%)	
Treatment Naïve	6 (10)
Transitioned from another IVIg product, n (%)	51 (85)
IVIG Liquid 10%^15^	34 (67)
IVIG Infusion 10%^16^	10 (20)
IVIG Liquid 5%^17^	4 (8)
IVIG 10% Liquid^18^	2 (4)
IVIG, unspecified	1 (2)
Transitioned from SCIg product, n (%)	3 (5)
facilitated SCIg Infusion 10%, (HyQvia)^19^	1 (33)
SCIg 20%^20^	2 (67)

*Cardiac comorbidities included: arrhythmias (n=9), coronary artery disease (n=7), valvular disease(n=6), congestive heart failure (n=3), myocardial infarction (n=2), and cardiomyopathy (n=2).

a Octapharma 10% (Octapharma USA Inc.).

b GAMMAGARD LIQUID (Baxalta US Inc.).

c Octapharma 5% (Octapharma USA Inc).

d PRIVIGEN (CSL Behring AG).

e HYQVIA (Baxalta US Inc.).

f HIZENTRA^®^ (CSL Behring AG).

Of the cohort, 54 patients (90%) were Ig-treatment experienced (IVIG or SCIG) and 6 (10%) were naïve to Ig replacement therapy. Of Ig treatment-experienced patients, 51 (85%) patients transitioned to BIVIGAM from another IVIG product with 3 (5%) transitioning from subcutaneous immune globulin (SCIG) to BIVIGAM. Those switching from another product had been on Ig therapy for a median of 2.5 years (min 0.1, max 8.6).

Over the course of the study, 346 BIVIGAM infusions were administered. **Table 2** provides BIVIGAM treatment, dosing and administration details. Patients received a mean ± SD initial dose of 492 mg/kg ± 200.8 and received a mean of 5.8 infusions ± 2.6. High-dose IVIG was observed in the patient with dermatomyositis (n = 1), who received IVIG at 2 g/kg divided over 2 days. Patients were dosed at frequencies of every 2 weeks (n = 1, 2%), 3 weeks (n = 7, 12%), 4 weeks (n = 51, 85%), or 6 weeks (n = 1, 2%). Infusion rates were titrated upward over a mean ± SD of 80 ± 27.9 minutes to a final mean maximum infusion rate of 158 ± 28.8 mL/hr. Maximum infusion rates ranged from 100 to 280 mL/hr, though most infusions 271 (78%) were given at a maximum rate of 150 mL/hr per standardized protocol.

**Table 2:** BIVIGAM dosing and administration.

Parameter	All infusions N=346 infusions
BIVIGAM dosing	
Initial dose in mg/kg, mean ± SD	492 ± 200.8
Initial dosing interval	
Every 3 weeks, n (%)	47 (14)
Every 4 weeks, n (%)	293 (85)
Other, n (%)^a^	6 (1)
Number of infusions per patient, mean ± SD	5.8 ± 2.6
BIVIGAM administration	
Infusion ramping time in minutes, mean ± SD	80 ± 27.9
Maximum infusion rate in mL/hr, mean ± SD	158 ± 28.8
Infusion rate range, mL/hr	100-280
Infusions administered at standard protocol rate, n (%)	271 (78)

a Other includes every 2 weeks (n=5) and every 6 weeks (n=1).

The use of medications prior to BIVIGAM infusions (or premedication) commonly occurred during the study period per standard practice in most physician office infusion centers. (**Table 3**). Fifty patients (83%) received premedications prior to 266 infusions. The most common premedication utilized by study patients was diphenhydramine (67% of patients, n=40) with 207 infusions, followed by acetaminophen with 194 infusions (65% of patients, n=39), corticosteroids with 145 infusions (45% of patients, n=27), and other premedications with 30 infusions (13% of patients, n=8).

**Table 3:** Pre-medications and hydration administered with BIVIGAM.

Parameter	All infusions N=346 infusions
Infusions with pre-medication, n (%)	266 (77)
Pre-medications per infusion, mean ± SD	2.0 ± 0.9
Pre-medications	
Diphenhydramine, n (%)	207 (60)
Acetaminophen, n (%)	194 (56)
Corticosteroids, n (%)^a^	145 (42)
Other, n (%)^b^	30 (9)
Hydration, n (%)	
Hydration Administered	159 (46)
Pre-infusion hydration	
Infusions with 0.9% sodium chloride, n (%)	100 (29)
Infusion volume in mL, mean ± SD	383 ± 196.1
Post-infusion hydration	
Infusions with 0.9% sodium chloride, n (%)	118 (34)
Infusion volume in mL, mean ± SD	198 ± 120.5

a Includes hydrocortisone (n=68 infusions), methylprednisolone (n=52), dexamethasone (n=24), prednisone (n=1).

b Other includes ondansetron (n=13), ibuprofen (n=8), famotidine (n=4), loratadine (n=3), hydrocodone with acetaminophen (n=1), levocetirizine (n=1).

More than half of the patients (55%, n=33) received no hydration before or after BIVIGAM. The remainder received hydration with 0.9% sodium chloride in 159 infusions of BIVIGAM. (**Table 3**). A total of 27 patients (45%) received pre-infusion hydration, post-infusion hydration, or both during the study period. Twenty-eight percent of patients (n=17) received pre-infusion hydration prior to IVIG at volumes of 250 mL (n=11), 500 mL (n=6), or 1000 mL (n=1). Post-infusion hydration was administered in 35% of patients (n=21), where hydration volumes of 100 mL (n=10), 250 mL (n=9), or 500 mL (n=3) were observed over 118 infusions. Some patients (18%, n=11) received both pre-infusion and post-infusion hydration with 0.9% sodium chloride.

**Figure 1:**
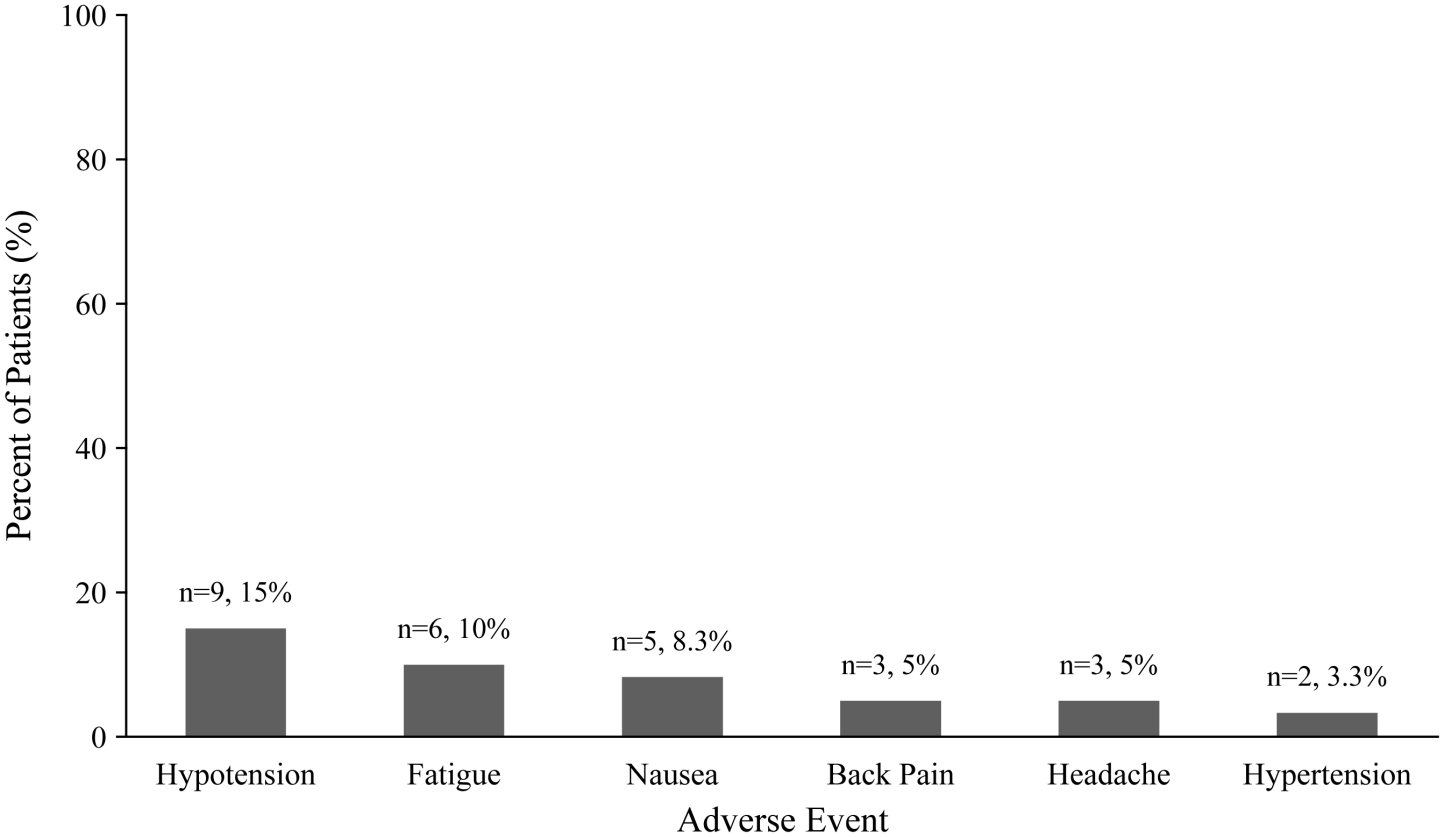
Common adverse events.

Over the course of the study, 46 AEs were reported in 23 patients (38.3%). **Figure 1** displays common AEs. The most common AE in patients was hypotension, occurring in 15% of patients. Other AEs included fatigue (10%), nausea (8%), back pain (5%), headache (5%), and hypertension (3%). Two infusions were temporarily interrupted due to AEs (one due to hypertension at the first infusion one for back pain at infusion 2, but both infusions subsequently resumed and were completed. Five patients discontinued treatment due to the AEs including nausea (n=2), hypertension (n=1), fatigue (n=1), and headache and aphthous ulcers (n=1). The overall rate of AEs per infusion was 0.13, calculated by dividing the total number of AEs (46) by the total number of infusions administered (346).

**Figure 2:**
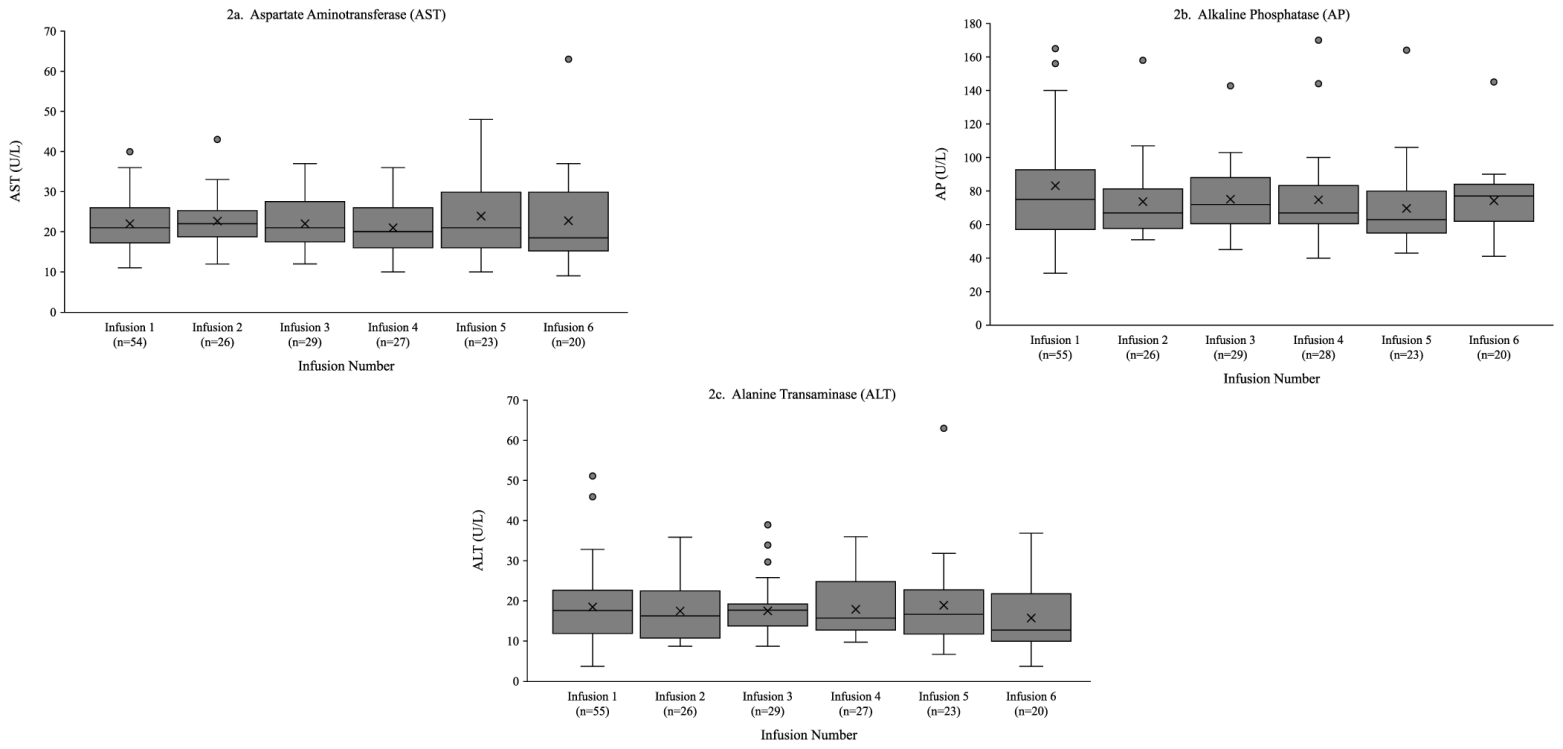
Liver function results over six infusions. Boxes represent the interquartile range with the horizontal line showing the median, whiskers indicate data range, circles denote outliers and “x” marks the mean.

Liver function results for AST, AP, and ALT are illustrated in **Figure 2**. For AST, 45 of 56 patients (80%) maintained normal levels throughout the study period. Eleven patients had elevated AST levels, where values normalized (n=3), fluctuated (n=5), or remained elevated (n=3) over the study period. For AP, 46 of 56 patients (82%) maintained normal levels. Eight patients had elevated values which normalized (n=2), fluctuated (n=2) or remained elevated (n=4) over the study period. Two patients had lower than normal AP levels. For ALT, 47 of 56 patients (84%) maintained normal levels. Nine patients had elevated levels which normalized (n=2), fluctuated (n=5), were elevated value at baseline only (n=1), or increased (n=1) over time.

Clinical laboratory values and blood pressure measurements before and after BIVIGAM treatment are summarized in **Table 4**. Laboratory follow-up values reflect the last measurement available for each patient, obtained during infusion 4,5, or 6. Overall, there were no statistically or clinically significant changes observed in laboratory indices over the study period (p > 0.05 for all). Hemoglobin, hematocrit, WBC and renal markers (BUN and SCr) remained largely stable, within minimal mean changes (≤0.3 units). Blood pressure exhibited a modest rise, within median systolic increasing from 127 [IQR:114-136] to 132[121-143] mmHg, and diastolic from 69 [62-77] to 73 [66-79] mmHg (p < 0.001 for both). While statistically significant, these changes were small and not clinically concerning. Post-infusion values remained within acceptable physiological limits.

**Table 4:** Changes in laboratory parameters and blood pressure.

Parameter	IVIG 10%			P-value
Laboratory values	Baseline n=40	Follow-up^a^ n=40	Mean change n=40	
Hg, g/dL, mean ± SD	12.7 ± 1.3	12.6 ± 1.5	-0.1	0.58
Hct, % of RBC, mean ± SD	38.2 ± 3.2	38.1 ± 4.2	-0.1	0.82
WBC, 10^3^/mm^3^, mean ± SD	6.6 ± 2.0	6.8 ± 1.9	0.2	0.47
BUN, mg/dL, mean ± SD	18.2 ± 6.0	17.9 ± 5.5	-0.3	0.69
SCr, mg/dL, mean ± SD	1.1 ± 1.1	0.9 ± 0.3	-0.2	0.4
Blood Pressure	Pre-infusion n=249	Post-infusion n=249	Mean change n=249	P-value
Systolic in mmHg, median [IQR]	127 [114 - 136]	132 [121 - 143]	6 ± 17.4	<0.001
Diastolic in mmHg, median [IQR]	69 [62 - 77]	73 [66 - 79]	3 ± 9.8	<0.001

Hg, hemoglobin; Hct, hematocrit; WBC, white blood cell; BUN, blood urea nitrogen; SCr, serum creatinine, SD, standard deviation; IQR, interquartile range.

a Last follow-up laboratory value available from infusion 4, 5, or 6.

## Discussion

We present outcomes in patients who infused BIVIGAM at outpatient physician office infusion centers over a six-month time period. A total of 60 patients received an average of 5.8 infusions. Patients were mostly female, Ig treatment-experienced, and had a primary diagnosis of PID. One patient received BIVIGAM for dermatomyositis and one for CLL. Patients utilized dosing and treatment intervals consistent with literature and prescribing information (9).

The use of prior Ig therapy was common among study patients (90%), whereas 10% were treatment naïve. Most patients transitioned to BIVIGAM from alternative intravenous Ig therapies and three patients transitioned from subcutaneous Ig therapy immediately prior to treatment initiation. The previous clinical trial with BIVIGAM only included patients who transitioned from other IVIG therapies (9). Our data is unique in that it adds to the literature by providing real-world evidence for the use of BIVIGAM in patients transitioning from subcutaneous Ig replacement therapy.

Most study patients received infusions at the standard infusion center protocol rate of 150 mL/hr, which is lower than the maximum recommended rate provided in the prescribing information (9). Pre-medications and 0.9% sodium chloride hydration were also administered per prescriber discretion. Over three-fourths of patients received pre-medications and received hydration with sodium chloride 0.9%. This was based upon each provider’s discretion and upon consistent with practice protocols for all immunoglobulin therapies.

The majority of patients (80%) completed six months of BIVIGAM treatment. The most common cause of discontinuation was due to AEs. Although the rate of discontinuation due to AEs was higher than the 2% rate reported in the clinical trial, our overall rate of AEs per infusion at 0.13 per person per year was lower than that reported in the clinical trial at 0.58, which was reported prior to the manufacturing optimization of BIVIGAM (9).

Liver function tests (AST, ALT, AP) were generally stable throughout the study with elevations mostly occurring at baseline. No patients required therapy modifications. There were no clinically relevant changes in hemolytic or renal function laboratory values across six months of therapy. A small but statistically significant increase in blood pressure was observed following infusions. However, these changes were clinically insignificant and did not indicate any pathologic elevation.

This was a real-world, retrospective, observational study, where inherent limitations existed. Limitations include selection of patients due to center recruitment, data availability, missing data, heterogeneous physician-driven infusion practices, and retrospective nature of nature of the study. Some laboratory and infusion data were missing at later timepoints. These missing data were not imputed and are displayed as observed (e.g., **Figure 2**), which may have introduced bias but reflects real-world clinical practice. Although all data available was utilized for this study, sample size remained at 60 total patients, limiting statistical analysis. These limitations may reduce generalizability and statistical power, therefore caution must be exercised when interpreting these findings or extending them to broader populations such as non-US populations, wider PID populations, or other infusion settings, as these populations were not studied. To address these limitations, future real-world studies employing a larger sample sizes and with longer follow-up periods are warranted to confirm durability of tolerability and safety outcomes.

Overall, this study has many strengths. It represents the largest retrospective, observational study of BIVIGAM since undergoing its optimized manufacturing process. Secondly, the uniqueness of the data cannot be disputed. The physician office infusion centers represented a geographically diverse group of patients receiving BIVIGAM. Finally, this study fills a gap in the literature and provides real-world evidence on the tolerability of BIVIGAM for future prescriber and payor decision-making.

In summary, BIVIGAM was well-tolerated within this real-world outpatient setting. Physician office infusion centers with trained nurses and physician oversight serve as an effective setting for the management and treatment of patients. BIVIGAM therapy demonstrated safety and tolerability in patients, and adverse events were lower than reported in the clinical trial. Standardized protocols, slower than allowed infusion rates, and an optimized manufacturing process of BIVIGAM may have contributed to its favorable tolerability.

## Data Availability

The datasets presented in this article are not readily available because the dataset will not be shared externally. Requests to access the datasets should be directed to Lucinda Van Anglen; Lvananglen@healix.net.
